# Optimal Brain MRI Protocol for New Neurological Complaint

**DOI:** 10.1371/journal.pone.0110803

**Published:** 2014-10-24

**Authors:** William A. Mehan, R. Gilberto González, Bradley R. Buchbinder, John W. Chen, William A. Copen, Rajiv Gupta, Joshua A. Hirsch, George J. Hunter, Scott Hunter, Jason M. Johnson, Hillary R. Kelly, Mykol Larvie, Michael H. Lev, Stuart R. Pomerantz, Otto Rapalino, Sandra Rincon, Javier M. Romero, Pamela W. Schaefer, Vinil Shah

**Affiliations:** Neuroradiology Division, Massachusetts General Hospital, Harvard Medical School, Boston, Massachusetts, United States of America; University of Ulm, Germany

## Abstract

**Background/Purpose:**

Patients with neurologic complaints are imaged with MRI protocols that may include many pulse sequences. It has not been documented which sequences are essential. We assessed the diagnostic accuracy of a limited number of sequences in patients with new neurologic complaints.

**Methods:**

996 consecutive brain MRI studies from patients with new neurological complaints were divided into 2 groups. In group 1, reviewers used a 3-sequence set that included sagittal T1-weighted, axial T2-weighted fluid-attenuated inversion recovery, and axial diffusion-weighted images. Subsequently, another group of studies were reviewed using axial susceptibility-weighted images in addition to the 3 sequences. The reference standard was the study's official report. Discrepancies between the limited sequence review and the reference standard including Level I findings (that may require immediate change in patient management) were identified.

**Results:**

There were 84 major findings in 497 studies in group 1 with 21 not identified in the limited sequence evaluations: 12 enhancing lesions and 3 vascular abnormalities identified on MR angiography. The 3-sequence set did not reveal microhemorrhagic foci in 15 of 19 studies. There were 117 major findings in 499 studies in group 2 with 19 not identified on the 4-sequence set: 17 enhancing lesions and 2 vascular lesions identified on angiography. All 87 Level I findings were identified using limited sequence (56 acute infarcts, 16 hemorrhages, and 15 mass lesions).

**Conclusion:**

A 4-pulse sequence brain MRI study is sufficient to evaluate patients with a new neurological complaint except when contrast or angiography is indicated.

## Introduction

Diagnostic medical imaging is one of the major driving forces behind rising healthcare expenditures. Between 2000 and 2006, Medicare spending on imaging services more than doubled, increasing to $14 billion^1^. The volume of brain MR studies performed may continue to rise in the future due to the expansion of health care insurance under the Patient Protection and Affordable Care Act (PPACA) and as a result of an aging population. At our institution, patients with various neurologic complaints are often imaged with MRI protocols that may involve a large number of pulse sequences, resulting in long scan times. There have been major advances in MRI hardware and software technology since the days of the first nuclear magnetic resonance (NMR) images in 1974. These advancements have provided us with an array of available MR sequences to choose from for our neuroimaging protocols. Long scan times related to multiple sequence acquisitions likely result in patient inconvenience and discomfort. It is unclear whether the multiple MR sequences performed are necessary and which of the sequences add value to the interpretation of the study. Optimizing the number of sequences in a routine screening brain MR protocol could improve efficiency as well as increase patient compliance and satisfaction by reducing scan time. Our goal was to investigate the diagnostic accuracy of brain MRI protocols containing a limited number of basic sequences in the evaluation of patients with new neurologic complaints.

## Methods

### Patient Selection

This retrospective study was approved by the Partners Human Research Committee, our hospital's Institutional Review Board (IRB). Consent was not obtained and patient records/information was anonymized and de-identified prior to analysis.

We performed a retrospective study of consecutive brain MR examinations performed at our institution, a major urban academic medical center, between February 20, 2010 and April 21, 2010. Studies included patients of all ages who underwent brain MR imaging for the evaluation of new neurologic complaints for which there was no established diagnosis stated on the radiology requisition. We included studies referred from the emergency department, inpatient and outpatient settings. Both intravenous contrast- enhanced and unenhanced studies were included as were studies that contained MR angiogram (MRA) and MR venogram (MRV) sequences. Image review commenced in June 2012, at least 24 months after the examinations were performed. Exclusion criteria were MR studies performed for follow-up evaluation of pre-existing neurological conditions (e.g. serial neuro-oncology studies) and specialized MR protocols (e.g. pituitary, orbits, brainstem and internal auditory canal studies).

All MRI examinations were performed on either 1.5T (Tesla) or 3T MR systems (GE Healthcare Clinical Systems, Wauwatosa, WI, USA; Siemens Medical Solutions, Erlangen, Germany). A total of 9 MRI scanners were used during the period that was reviewed. Eight scanners had field strengths of 1.5T. Six were manufactured by General Electric; 3 were located within the hospital and 3 were located at free standing imaging centers. Two 1.5T MRI scanners were manufactured by Siemens and were located within the hospital. Additionally, a single Siemens 3T scanner was located within the hospital. All MR images in the study were reviewed on a PACS system (Agfa Impax 5.3, Agfa Healthcare, Mortsel, Belgium).

### Study Interpretation

The total number of consecutive MRI studies was partitioned into two separate data sets (group 1, MR studies from February 20, 2010 through March 23, 2010; group 2, MR studies from March 24, 2010 through April 21, 2010).

The group 1 neuroradiologists reviewed a three-sequence abbreviated image set composed of sagittal T1-weighted images, axial T2-weighted FLAIR images, and images derived from a single diffusion-weighted imaging sequence, which included images with and without diffusion gradients, as well as derived apparent diffusion coefficient maps. The neuroradiologists interpreted those three MRI sequences while blinded to both the additional MRI sequences that were acquired, and to the official radiologic report. For each case, a group 1 neuroradiologist formulated an impression based on the three limited sequences and then subsequently compared it to the unblinded impression in the reference official radiologic report. For any discrepancy between the two interpretations, the neuroradiologist was instructed to revisit the limited MR sequences and determine whether, in retrospect, the discrepant findings could have been made on the limited sequences, but were missed due to perceptual error.

The second phase of the study involved a different set of staff neuroradiologists with experience ranging from 2 to 20 years. The group 2 neuroradiologists reviewed a four-sequence abbreviated image set composed of the three basic sequences that were reviewed by the group 1 neuroradiologists, with the addition of an axial magnetic susceptibility sensitive sequence (either T2* gradient recalled echo (GRE), or susceptibility weighted imaging (SWI), including susceptibility weighted angiography (SWAN)). The neuroradiologists interpreted those four MRI sequences while blinded to both the additional MRI sequences that were acquired, and to the official radiologic report. For each case, a group 2 neuroradiologist formulated an impression based on the four limited sequences and then subsequently compared it to the unblinded impression in the official radiologic report, (the reference standard). For any discrepancy between the two interpretations, the neuroradiologist revisited the limited MR sequences and determined whether, in retrospect, the discrepant findings could have been appreciated on the limited sequences, but were missed due to perceptual error.

The limited sequence interpretations by the neuroradiologists in groups 1 and 2 were performed on the same PACS workstations as the original interpretations, and they are located in the same interpretation areas as in the original clinical interpretation. The only history that was permitted to the new study reviewer was the limited one provided with the original request for imaging. The neuroradiologists in groups 1 and 2 were allowed to review previous MRI studies when available, but only the limited MRI sequences on the prior study could be reviewed when formulating an impression. Readers in both groups were not permitted to review post-contrast, MRA, or MRV images, if these were acquired.

In order to eliminate recall bias, the assignment of MRI cases to each neuroradiologist was reviewed. If any case had been previously reviewed by the neuroradiologist to which the case was assigned, then the case was reassigned to an additional neuroradiologist (RGG) who reviewed the case with the same instructions.

We designated findings and discrepancies as major if they were likely to have a significant impact on patient management. Level 1 findings were designated as those requiring immediate communication at our institution: acute infarction, intracranial hemorrhage and unexpected mass lesion. The following imaging findings were also designated as major: subacute infarctions; posterior reversible encephalopathy syndrome (PRES); vascular malformations; malformations of cortical development (including focal cortical dysplasia); hydrocephalus; parenchymal lesions suspicious for infection, inflammatory disease, primary or secondary CNS neoplasm; and other mass lesions. Abnormalities of the major intracranial flow voids including arterial dissection, venous sinus thrombosis and intra-arterial thrombus were also considered major abnormalities. Acute/subacute infarctions required observation of decreased diffusivity in DWI sequences. Microhemorrhages (punctate parenchymal foci of susceptibility effect) were considered potentially important findings. Other typically incidental findings were designated as non-major.

### Statistical Analysis

A descriptive analysis of the data from groups 1 and 2 was performed.

## Results

Out of a total of ∼3750 brain MRI studies, 998 brain MRI studies met our eligibility criteria. The first 497 studies were placed in group 1, and the remaining 499 in group 2.

**Figure 1:**
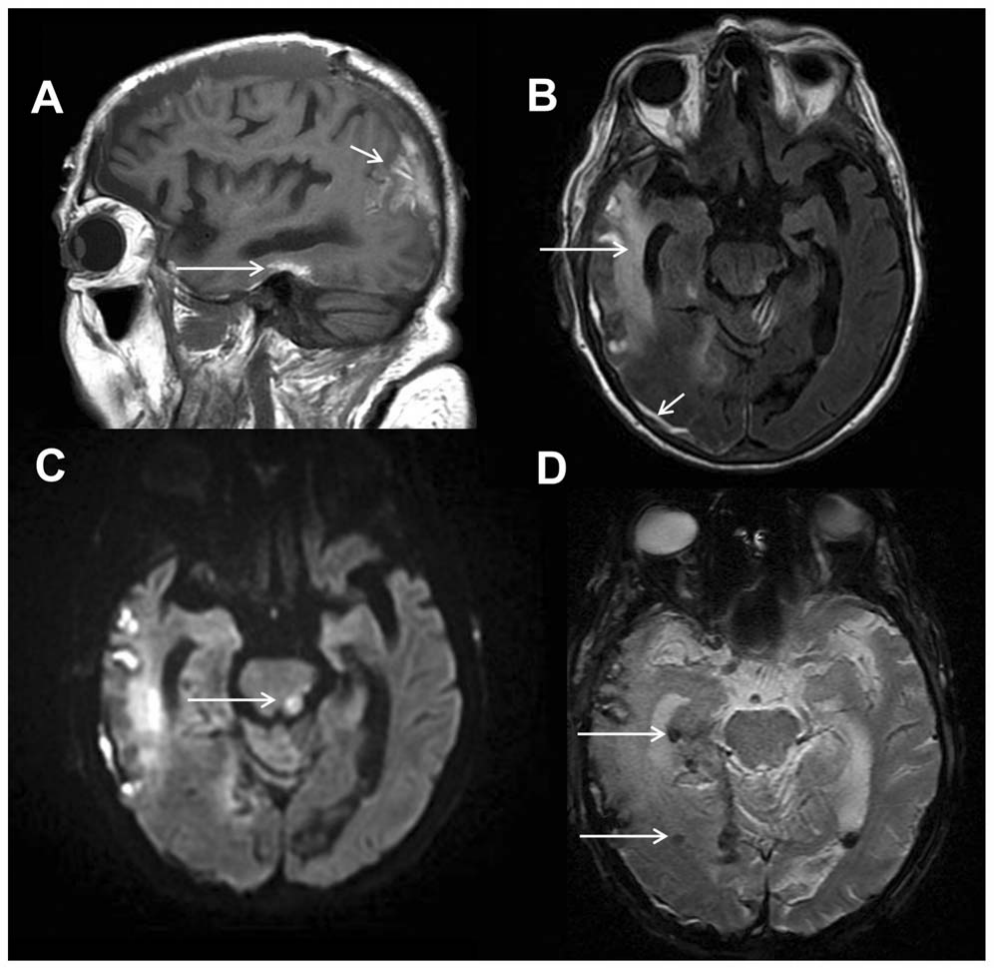
Intracranial hemorrhages and diffuse axonal injury. 83 year old female status post fall. Sagittal T1-weighted image (A) shows hyperintense signal involving the lateral right temporal lobe consistent with cortical contusion and subacute blood products (white arrow). Axial T2-weighted FLAIR image (B) depicts edema in the right temporal lobe from traumatic contusion (long white arrow) and a subdural hemorrhage overlying the lateral right temporal and occipital lobes (short white arrow). Axial diffusion weighted trace image (C) shows a focus of restricted diffusion within the left dorsolateral midbrain compatible with non-hemorrhagic diffuse axonal injury (white arrow). Axial gradient recalled echo image (D) shows a punctate focus of susceptibility effect within the right temporal lobe compatible with hemorrhagic diffuse axonal injury (white arrow).

**Figure 2:**
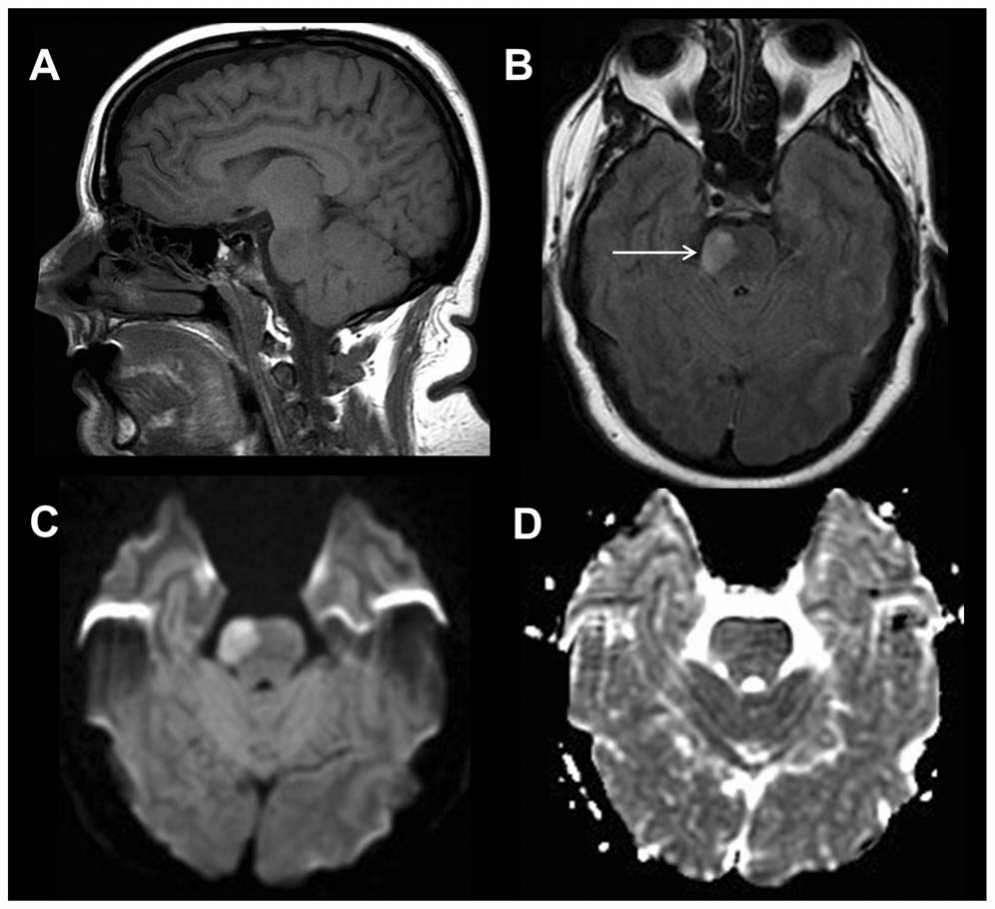
Brainstem mass lesion. 38 year old female with transient left sided weakness. Sagittal T1-weighted image (A) shows a subtle hypointense lesion within the pons. Axial T2-weighted FLAIR image (B) demonstrates a hyperintense, expansile mass-like lesion within the right mid pons (white arrow). Axial diffusion weighted trace (C) and apparent diffusion coefficient images (D) reveal slightly increased diffusivity within the lesion compatible with a demyelinating lesion, neoplasm, or subacute infarction.

In group 1, 84 major findings were identified (16.9% of studies, Table 1). All 41 level 1 findings (28 acute infarcts, 9 hemorrhages, and 4 mass lesions) were identified on the limited sequences. Examples of cases with accurately interpreted major findings included traumatic brain injury with multi-compartment intracranial hemorrhages (Figure 1) and neoplasms (Figure 2). Of these 84 major findings, there were 21 discrepancies between the interpretation based on the abbreviated MRI protocol, and the official interpretation from the radiologic report, yielding an error rate of 25.0%.

**Table 1:** Patient Demographics and MRI Findings Group 1.

Parameter	Group 1 (n = 497)	Group 2 (n = 499)
Age (years)	mean 54.3 (range 0.5–98)	mean 51.3 (range 0.5–93)
Male∶Female (%)	43∶57	38∶62
**Significant MRI Findings**	84	117 (23.4%)
Acute/subacute infarction	39 (46.4%)	67 (57.3%)
Intracranial hemorrhages	9 (10.7%)	7 (6.0%)
Posterior reversible encephalopathy syndrome (PRES)	2 (2.4%)	4 (3.4%)
Vascular lesions*	4 (4.8%)	4 (3.4%)
Cavernous malformations	4 (4.8%)	1(0.9%)
Active demyelinating lesions	3 (3.6%)	2(1.7%)
Hydrocephalus	1 (1.2%)	3 (2.6%)
Metastases	5 (6.0%)	9 (7.7%)
Meningiomas or schwannomas	8 (9.5%)	7 (6.0%)
Mass lesions**	4 (4.8%)	11 (9.4%)
Infectious/Inflammatory lesions	3 (3.6%)	2(1.7%)
Cortical dysplasia	2 (2.4%)	0
**Major discrepancies**	21	19 (16.2%)
Enhancing lesions***	12 (57.1%)	17 (89.5%)
*Punctate acute/subacute infarcts*	4 (19.0%)	0
Cortical dysplasias	2(9.5%)	0
Vascular lesions	3 (14.3%)	2 (10.5%)
**Microhemorrhage discrepancies**		
Microhemorrhages	Group 1 (n = 19)	Group 2 (n = 28)
Discrepancies	15 (77.8%)	0

* Venous sinus thrombosis, ICA dissection, arterial thrombus, absent vertebrobasilar flow voids, MCA branch thrombosis

** Cerebellar mass, brainstem mass, optic nerve sheath mass, thalamic masses, lobar masses, pituitary/sellar masses, corpus callosal mass, clival mass, colloid cysts

***Included probable metastases, probable primary tumors, inflammatory/demyelinating lesions, and indeterminate punctate enhancing foci

**Figure 3:**
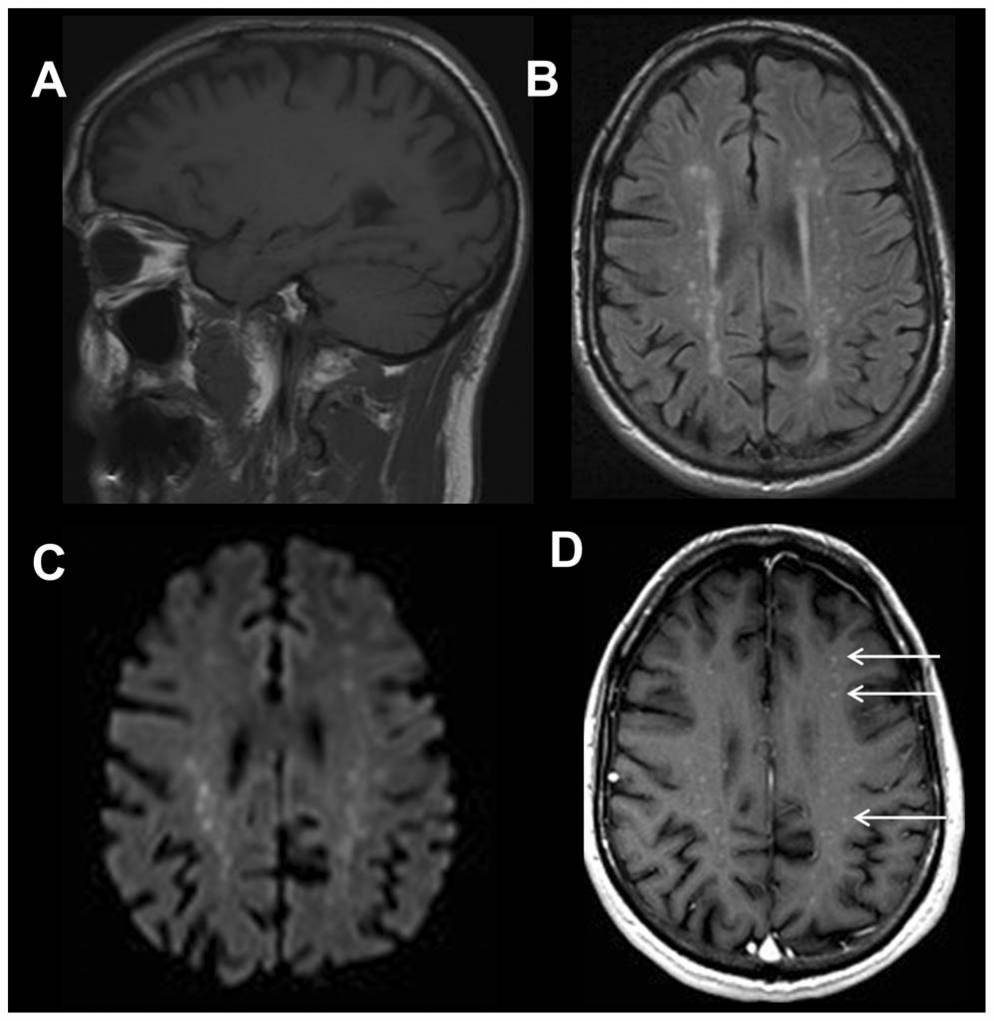
Foci of enhancement in a 57 year old male with ataxia. Sagittal T1-weighted image (A) shows no definite abnormality. Axial T2-weighted FLAIR image (B) demonstrates multiple punctate hyperintense foci involving the deep and periventricular white matter of the bilateral cerebral hemispheres with corresponding restricted diffusion on the axial diffusion weighted trace (C) image. Axial T1-weighted post contrast image (D) reveals numerous punctate foci of enhancement corresponding to the deep and periventricular white matter lesions (white arrows). In this patient, the differential diagnosis included intravascular lymphoma, granulomatous disease, neurosarcoidosis, and vasculitis.

**Figure 4:**
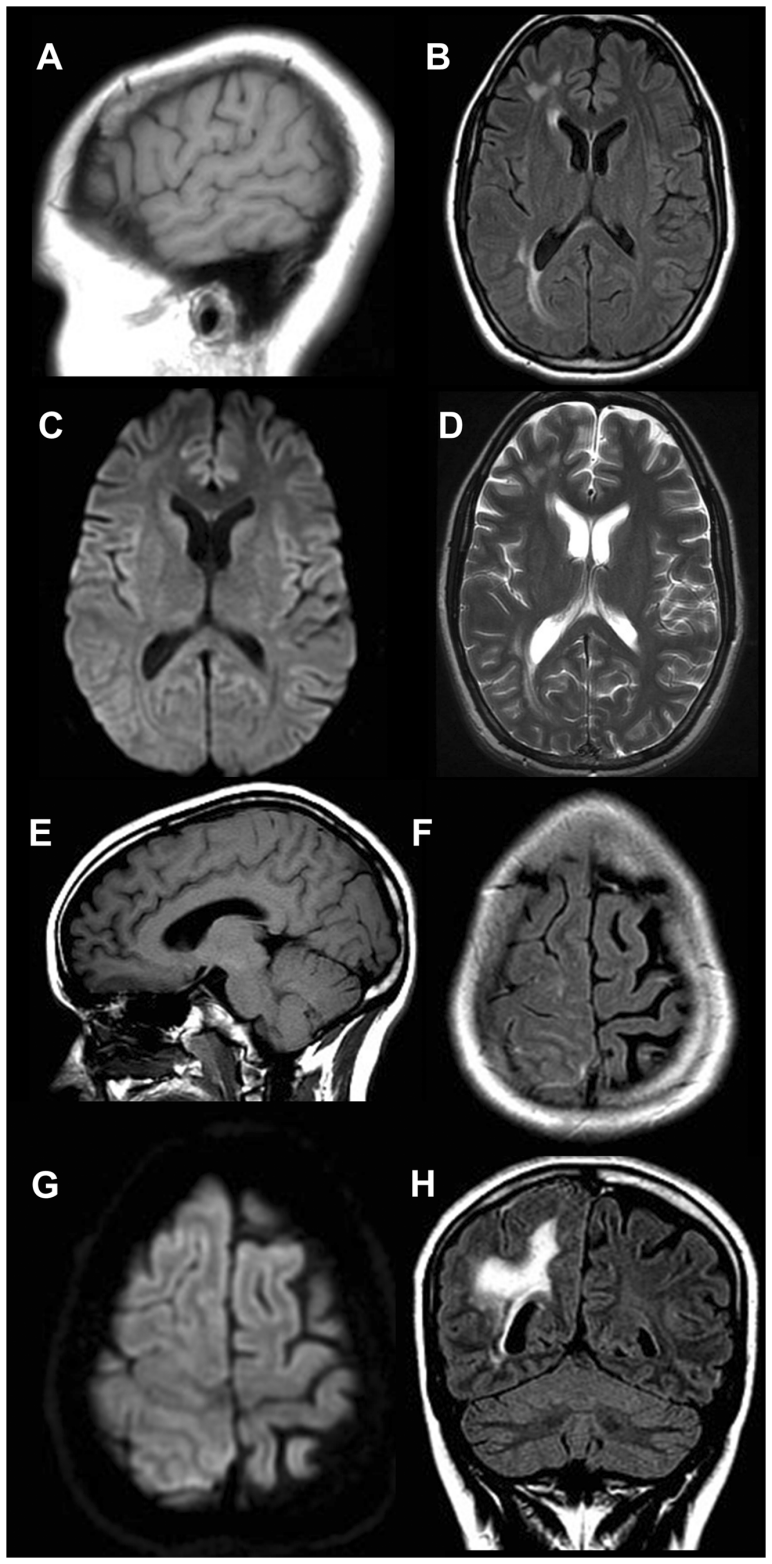
22 year old female with right frontal hemispheric dysplasia. Sagittal T1-weighted image (A) shows no definite abnormality. Axial FLAIR image (B) demonstrates subtle enlargement of the right hemisphere with focal areas of signal hyperintensity within the subcortical white matter of the right frontal and parietal lobes but without corresponding restricted diffusion on the axial diffusion weighted trace (C) image. Additionally noted is ipsilateral ventricular enlargement. Pachygyria was also demonstrated within the right frontal and parietal lobes, better demonstrated on more superior axial FLAIR images. Axial T2-weighted image (D) also demonstrates these findings. Sagittal T1-weighted image (E) shows no definite abnormality. Axial FLAIR image (F) demonstrates asymmetric cortical thickening, gyral enlargement within the right frontal and parietal lobes without restricted diffusion on the axial diffusion weighted trace (G) image, consistent with pachygyria. Coronal FLAIR image (H) reveals asymmetric cortical thickening, abnormal sulcation and white matter signal hyperintensity in the right parietal lobe. The group 1 reader described the right frontal lobe signal abnormality, but did not diagnose the full extent of the hemispheric abnormality. These findings are more apparent on the coronal FLAIR sequence, not available to the reader.

**Figure 5:**
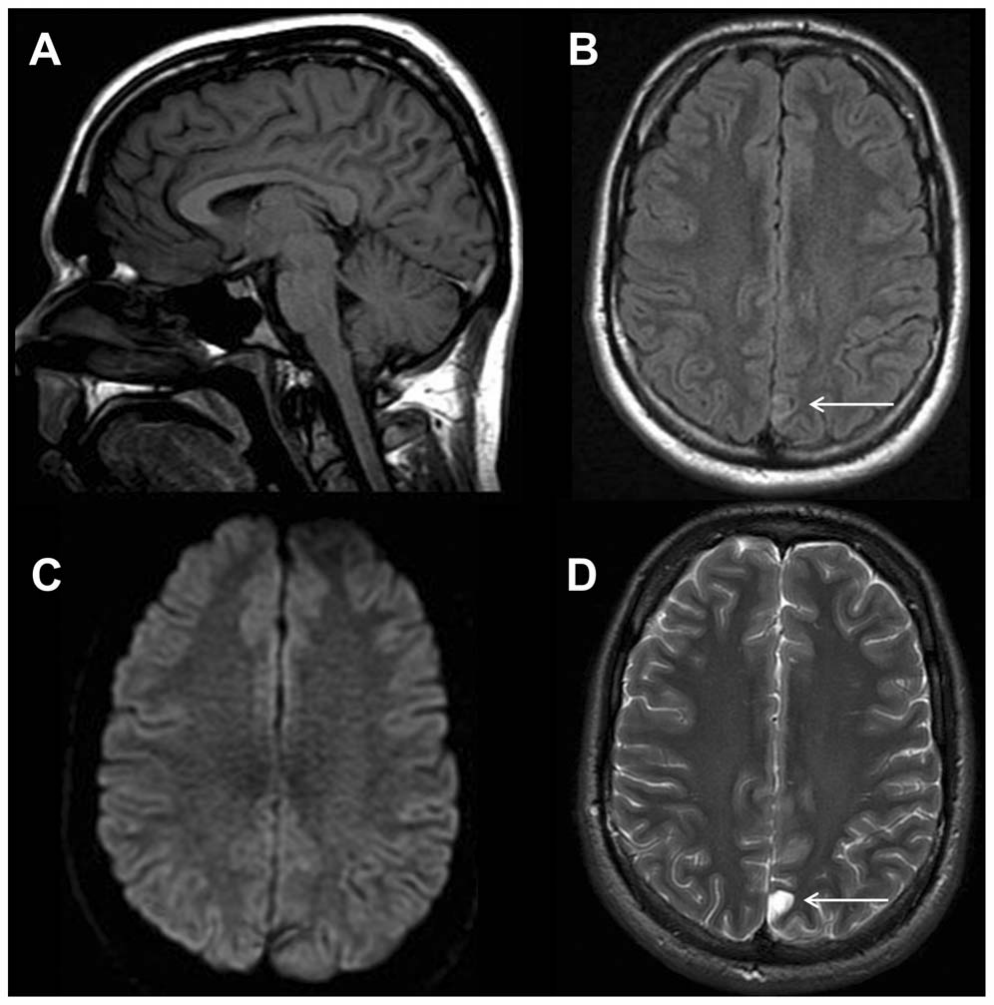
Cortical dysplasia versus dysembryoplastic neuroepithelial tumor in an 18 year old male with seizures. Sagittal T1-weighted image (A) shows no definite abnormality. Axial T2-weighted FLAIR image (B) demonstrates a subtle focal area of signal hyperintensity within the cortex and subcortical white matter of the left parafalcine parietal lobe(white arrow) with no corresponding restricted diffusion on the axial diffusion weighted trace (C) image. Axial T2-weighted image (D) again demonstrates the hyperintense lesion within the cortex and subcortical white matter of the left parafalcine parietal lobe (white arrow), which is compatible with cortical dysplasia or dysembryoplastic neuroepithelial tumor. This finding is subtle and was not seen prospectively when using the limited sequences. However, the lesion can be seen on the FLAIR images in retrospect. Furthermore, for seizure patients, additional sequences, including isotropic multiplanar sequences, could be added that would help in identifying subtle abnormalities.

The most common discrepant major findings were enhancing intracranial lesions (12 cases) (Figure 3). There were 4 small acute or subacute infarctions that were missed by two of the group 1 readers. All of these abnormalities were identified on the diffusion MR sequences when the readers re-examined these studies. Thus, these discrepancies were due to perceptual error. The diagnosis of cortical dysplasia was not made in 2 studies by group 1 readers. Abnormalities on the limited sequences were identified (Figure 4), but the diagnosis was not suggested. In the other case, a small non-enhancing lesion was only observed upon repeat review of the study (Figures 5).

There were 3 vascular abnormalities that were not identified on the abbreviated sequences; 2 cases of middle cerebral artery branch (MCA) thrombus in patients with concurrent acute infarctions and a case of transverse sinus thrombosis. The cases of MCA branch occlusion were not visualized on the 3-sequence series, although the acute infarctions in the MCA distribution were identified. The group 1 reader who did not identify the venous thrombosis reported that they would not have seen this lesion without an MR venogram.

**Figure 6:**
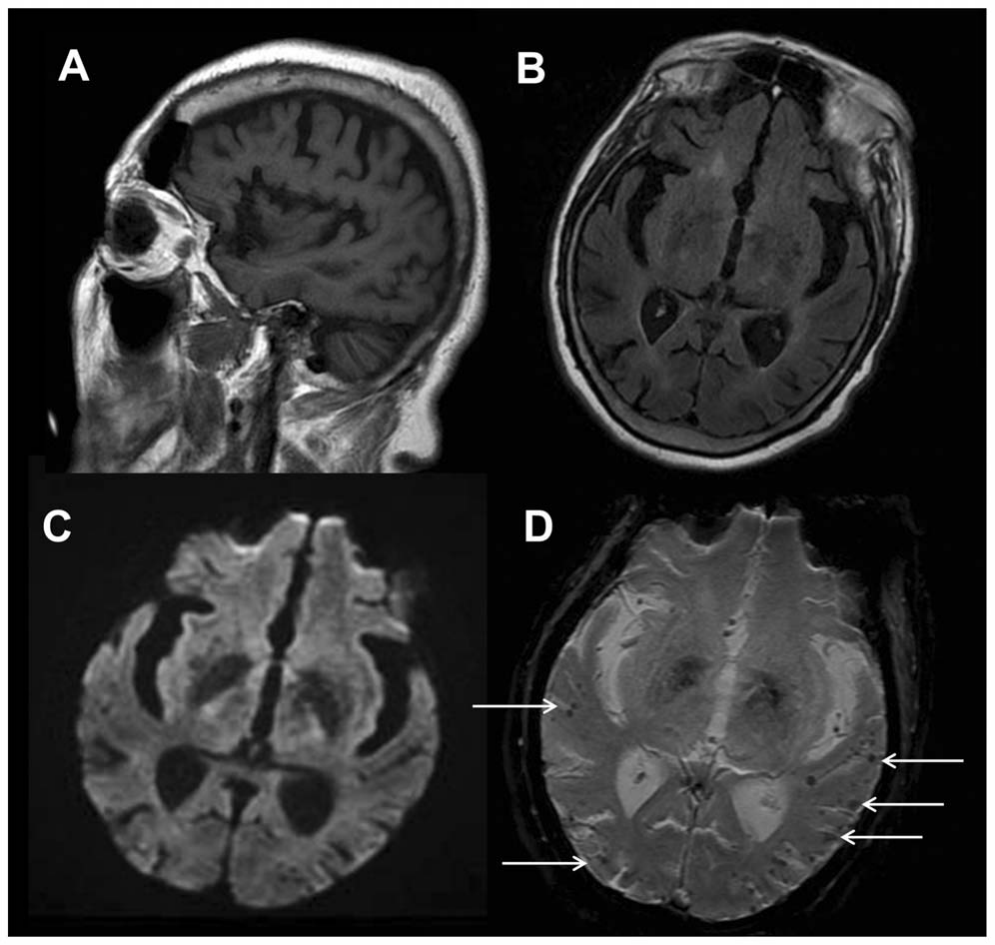
Cerebral amyloid angiopathy in a 78 year old female with altered mental status. Sagittal T1-weighted image (A) shows age appropriate parenchymal volume loss. Axial FLAIR image (B) demonstrates a small number of nonspecific hyperintense foci that are not unusual in a patient of this age. The axial diffusion weighted image (C) demonstrates no evidence of acute ischemia. Sequences A-C (group 1 sequences) show no definite abnormality that would explain the patient's symptoms. Axial gradient recalled echo image (D) reveals multiple punctate foci of susceptibility effect (white arrows) involving peripheral grey-white matter junctions, consistent with chronic microhemorrhages, which was not identified by the group 1 reader. This sequence alone (in addition to the other 3 sequences) provides the information needed to make the diagnosis of cerebral amyloid angiopathy.

In group 1, there were 19 cases that had evidence of probable microhemorrhage (3.8%). Out of these 19 cases, 15 were not identified by group 1 readers on the abbreviated MR sequences (77.8%) (Figure 6).

**Figure 7:**
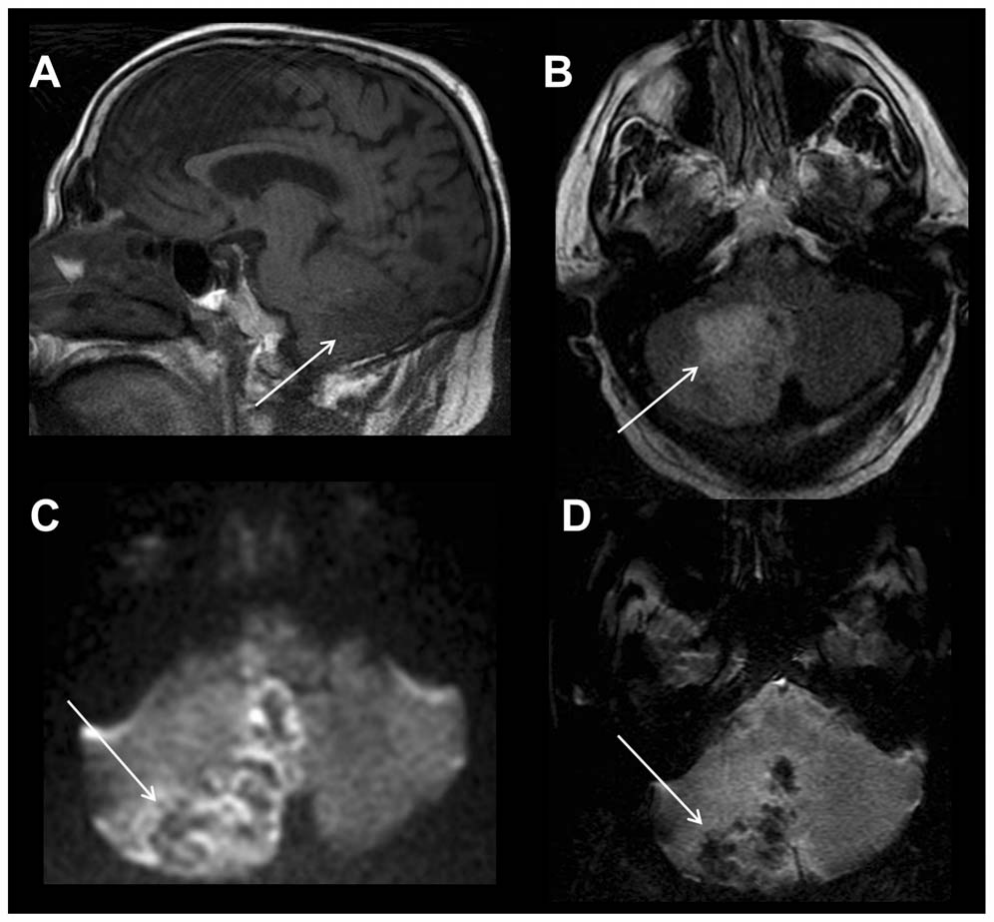
Hemorrhagic acute cerebellar infarction in a 79 year old male with a focal neurological deficit. Sagittal T1-weighted image (A) shows hypointense signal within the right inferior cerebellar hemisphere (white arrow). Axial T2-weighted FLAIR image (B) demonstrates geographic hyperintense signal and mass effect within the right inferior cerebellar hemisphere (white arrow). Axial diffusion weighted trace image (C) shows restricted diffusion in the right cerebellar hemisphere compatible with infarction (white arrow) as well as areas of hypointensity reflecting hemorrhage. Axial gradient recalled echo image shows susceptibility effect within the right cerebellar hemisphere, which is consistent with hemorrhage (white arrow).

**Figure 8:**
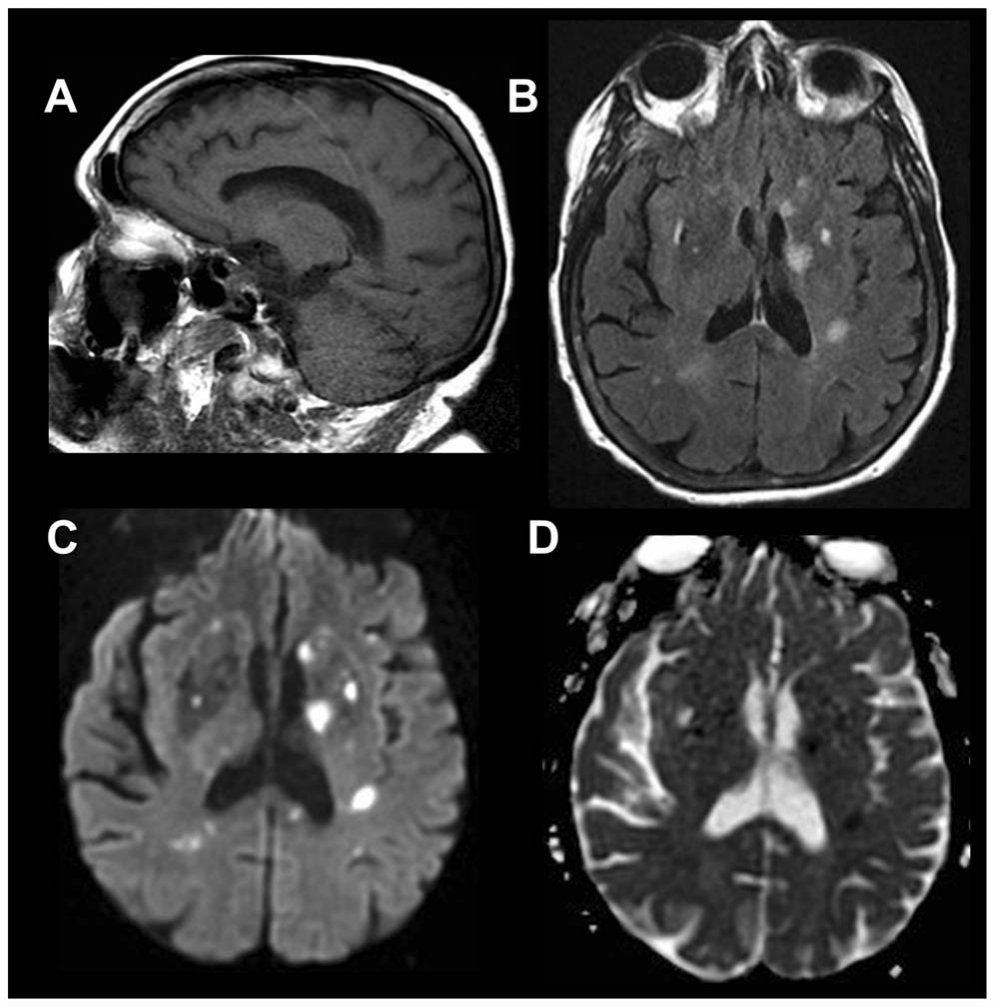
Acute infarctions in a 77 year old female with altered mental status. A 77-year-old woman underwent MRI examination because of altered mental status. Her complete examination included 12 pulse sequences, but all significant abnormalities are apparent in images derived from the three pulse sequences that comprised the abbreviated examination described in phase 1 of this study. A sagittal T1-weighted image (A) reveals no apparent abnormality. Axial T2-weighted FLAIR images (B) demonstrate multiple small hyperintense lesions in deep gray matter and white matter structures. The lesions are markedly hyperintense in diffusion-weighted images (C) and apparent diffusion coefficient maps derived from the DWI sequence (D) show restricted diffusion within the lesions, confirming that they are ischemic infarcts.

**Figure 9:**
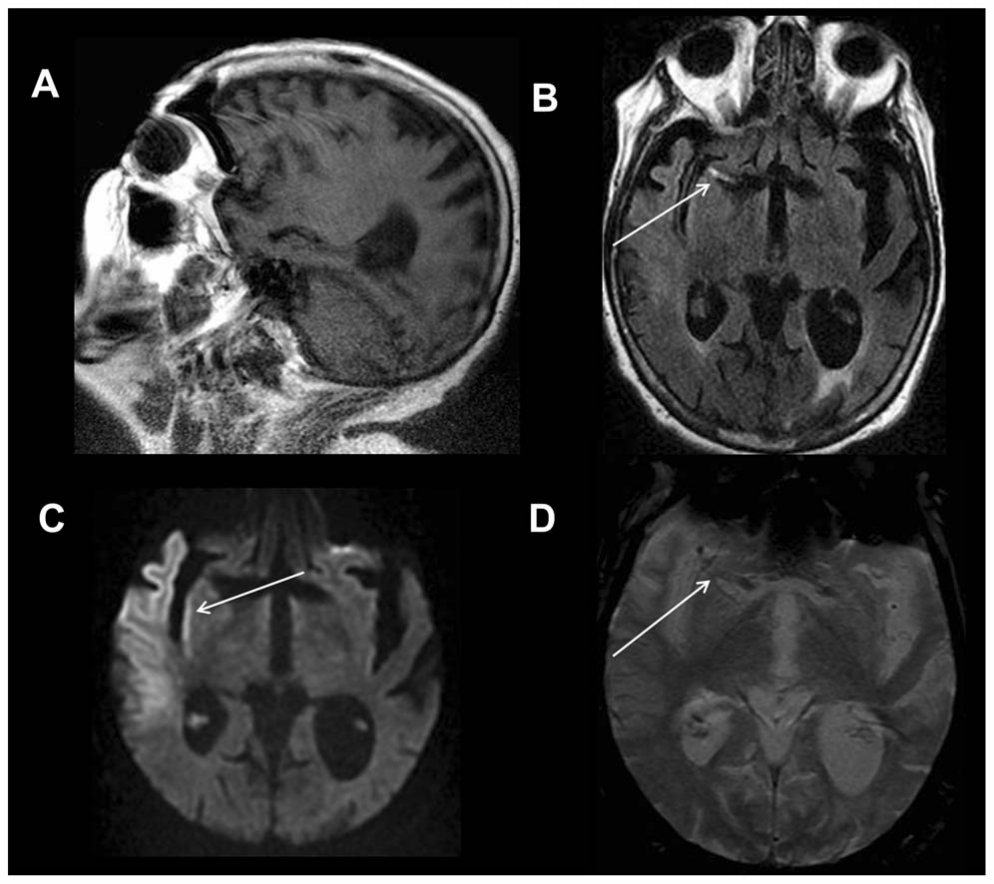
Acute stroke in a 78 year old female with altered mental status. Sagittal T1-weighted image of the right temporal lobe/insula (A) is normal. Axial T2-weighted FLAIR image (B) shows hyperintense signal within the M1 segment of the right middle cerebral artery (MCA), suggesting slow flow or clot (white arrow). Axial diffusion weighted image (DWI) (C) shows restricted diffusion within the right temporal lobe and insula, diagnostic of acute stroke to this region (white arrow). Axial gradient recalled echo (GRE) susceptibility image (D) shows subtle linear susceptibility effect within the right MCA, consistent with recent passage of embolic material (white arrow).

**Figure 10:**
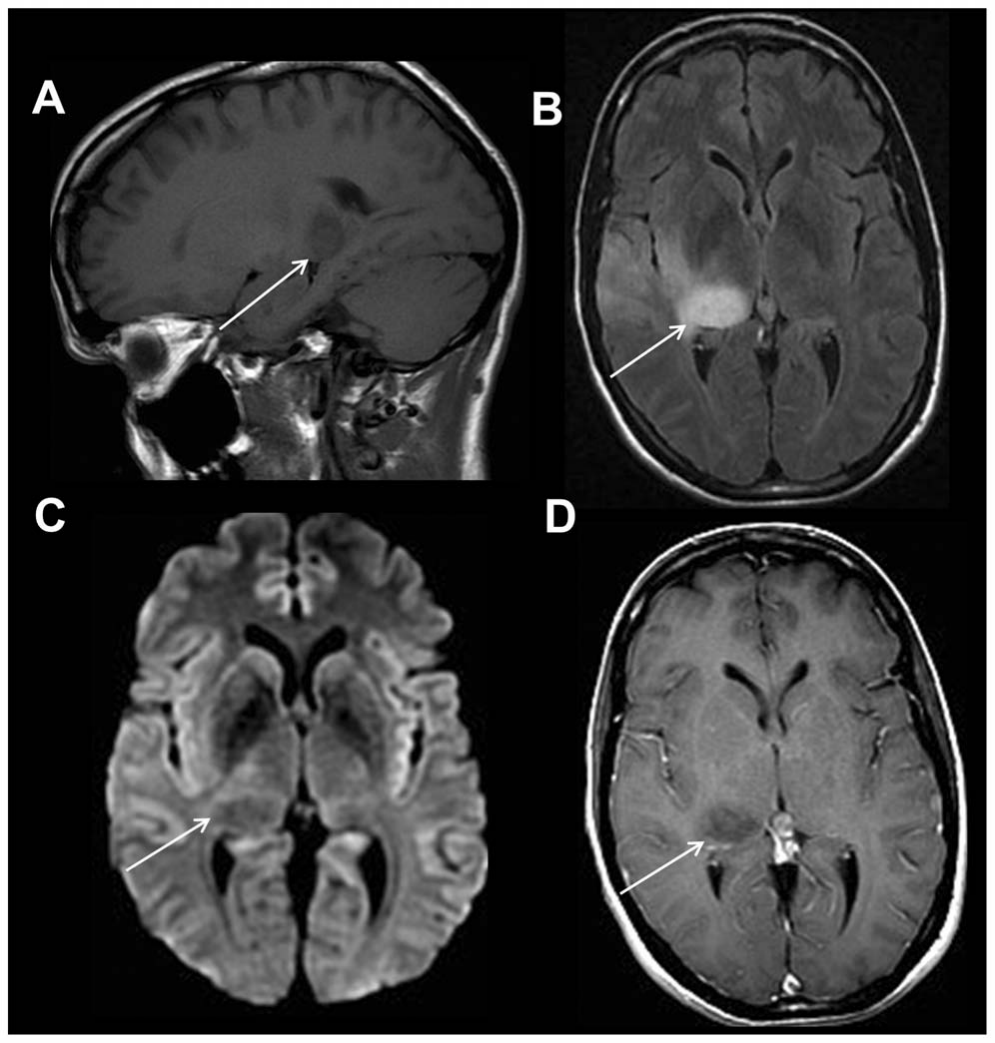
Right thalamic neoplasm identified in a 48 year old female with seizures. Limited pre-contrast set of imaging sequences are sufficient to clearly identify an expansile lesion within the pulvinar of the right thalamus; a finding requiring immediate communication for action (Level I) (white arrows). It is hypointense on sagittal T1-weighted image (A) and has associated hyperintensity on axial T2-weighted FLAIR extending into the adjacent insular-opercular region (B). Even within the confines of the limited set of sequences, neoplastic differential considerations are still able to be favored over acute ischemia as no evidence of restricted diffusion is seen on diffusion-weighted imaging (DWI) sequences; specifically low signal on DWI trace images (C) and elevated diffusivity on Apparent-Diffusion-Coefficient images (not shown). Post-contrast imaging, not initially available to the readers within the limited set of review sequences, demonstrated no abnormal enhancement (D).

**Figure 11:**
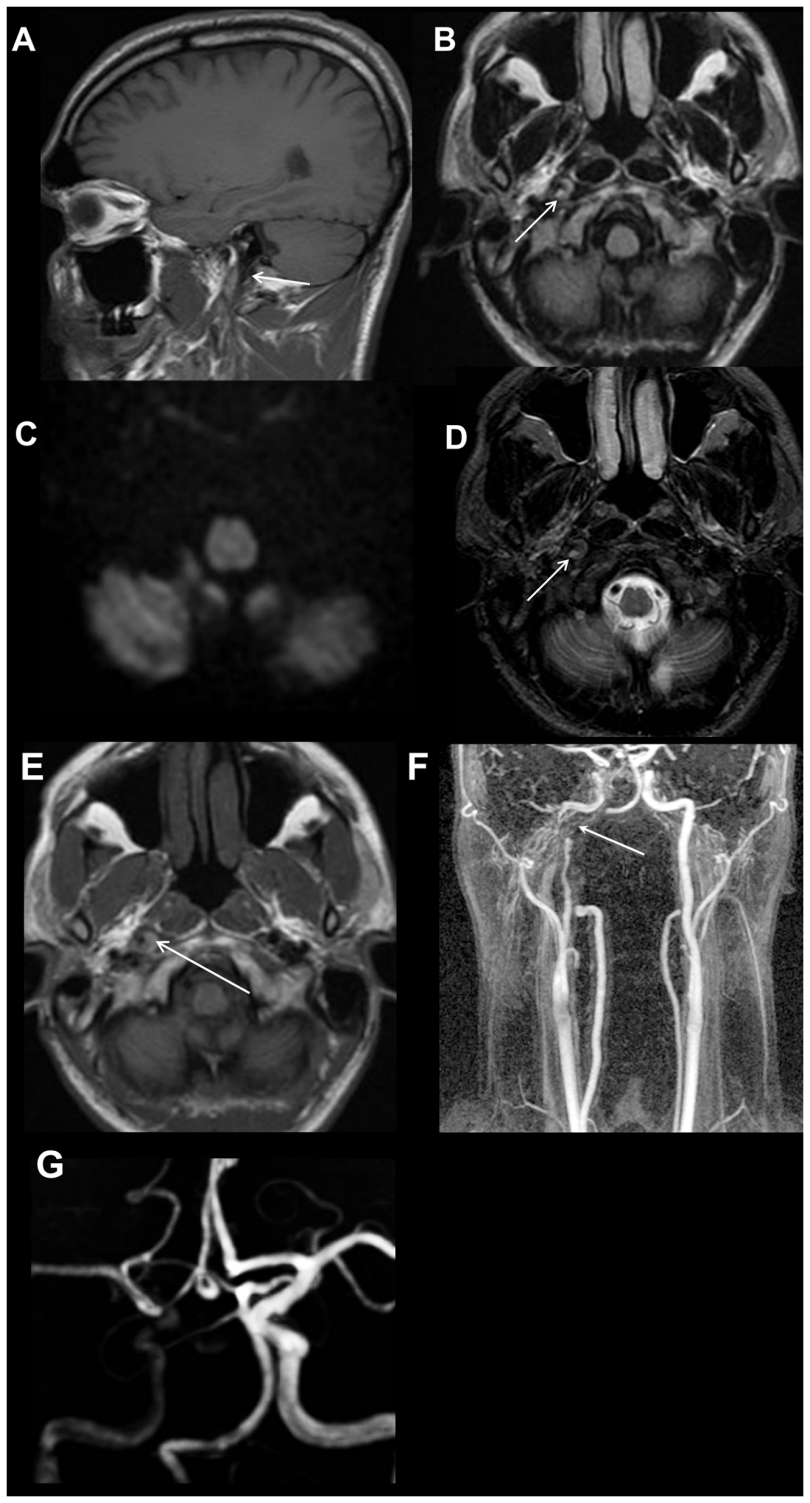
Internal carotid artery dissection in a 41 year old male with headache. The dissection was not identified on the limited 3 sequence review. Sagittal T1-weighted image (A) shows narrowing of the distal cervical portion of the right internal carotid artery with T1 hyperintense intramural hematoma (white arrow). Axial FLAIR image (B) demonstrates loss of the normal right internal carotid artery flow void (white arrow), which is more conspicuous on the axial T2-weighted image (D). Axial diffusion weighted trace image (C) shows no restricted diffusion in the region of the right internal carotid artery. Axial pre-contrast T1-weighted image (E) demonstrates an eccentric T1 hyperintense intramural hematoma (white arrow) in the distal cervical right internal carotid artery. MR angiography maximum intensity projection images reveal irregular critical narrowing (white arrow) of the distal cervical right ICA (F) and asymmetrical flow-related signal in the right intracranial ICA (G).

**Figure 12:**
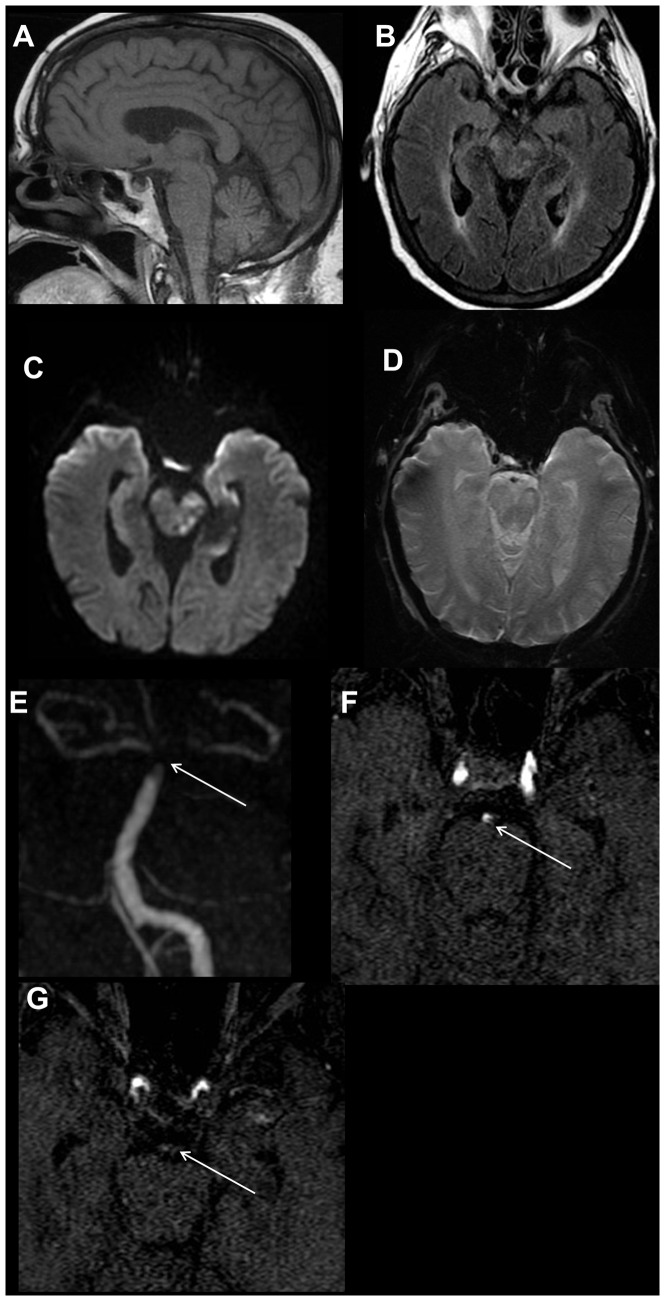
72-year-old female with altered mental status and ischemic stroke. On the 4-sequence MRI protocol (A-D), the axial FLAIR image (B) demonstrates multifocal ill-defined areas of signal hyperintensity within the midbrain with corresponding restricted diffusion on the axial diffusion weighted trace image (C), consistent with infarctions. Additional foci of infarction were seen in the left occipital lobe, left mesial temporal lobe, both thalami and the cerebellum (images not shown). No definite abnormality is seen on the sagittal T1-weighted image (A) or axial T2* image (D). MR angiography was also requested. MRA maximum intensity projection image (E) reveals thrombus with the distal basilar (white arrow) and proximal posterior cerebral arteries, which is confirmed on the axial source MR angiography images (F, G) (white arrow). Even in retrospect, this finding was not apparent on any of the sequences included in the 4-sequence MRI protocol. MRA imaging adds significant value in cases with clinical suspicion for vascular occlusion or thrombosis.

In the group 2 evaluation, there were 117 major findings in the 499 MR studies (23.4%, Table 2). Examples of cases with accurately detected major findings included early infarctions (Figures 7, 8, and 9) and neoplasms (Figure 10). Of these, 19 major findings were not identified on the abbreviated 4-sequence MRI series resulting in an error rate of 16.2%. Enhancing parenchymal lesions were the most commonly missed major findings (17 cases). The other major discrepancies were vascular lesions; one case of ICA dissection (Figure 11) and one case of vertebrobasilar arterial occlusion (Figure 12). In both of these cases, the readers reported that the absence of the flow voids was not identifiable without the aid of a non-inversion recovery T2-weighted sequence or MR angiogram (Figures 11D-F and 12E-G). There were 28 cases with probable microhemorrhages in the group 2 data set. All of these were identified by the group 2 readers.

**Table 2:** Patient Demographics and MRI Findings Group 2.

Parameter	Group 2 (n = 499)
Age (years)	mean 51.3 (range 0.5–93)
Male∶Female (%)	38∶62
**Significant MRI Findings**	117
Acute/subacute infarction	67 (57.3%)
Intracranial hemorrhages	7 (6.0%)
Posterior reversible encephalopathy syndrome (PRES)	4 (3.4%)
Vascular lesions*	4 (3.4%)
Cavernous malformations	1(0.9%)
Active demyelinating lesions	2(1.7%)
Hydrocephalus	3 (2.6%)
Metastases	9 (7.7%)
Meningiomas or schwannomas	7 (6.0%)
Mass lesions**	11 (9.4%)
Infectious/Inflammatory lesions	2(1.7%)
Cortical dysplasia	0
**Major discrepancies**	19
Enhancing lesions***	17 (89.5%)
*Punctate acute/subacute infarcts*	0
Cortical dysplasias	0
Vascular lesions	2 (10.5%)
**Microhemorrhage discrepancies**	
Microhemorrhages	Group 2 (n = 28)
Discrepancies	0

* Venous sinus thrombosis, ICA dissection, arterial thrombus, absent vertebrobasilar flow voids, MCA branch thrombosis

** Cerebellar mass, brainstem mass, optic nerve sheath mass, thalamic masses, lobar masses, pituitary/sellar masses, corpus callosal mass, clival mass, colloid cysts

***Included probable metastases, probable primary tumors, inflammatory/demyelinating lesions, and indeterminate punctate enhancing foci

## Discussion

Since the introduction of MRI for the clinical evaluation of brain pathology, a large number of pulse sequences have been developed, and those with the highest clinical value have been extensively evaluated. However, there is little documented research on the optimal set of MRI sequences for the evaluation of patients with specific clinical presentations. In this evaluation of MRI used in patients with a new neurological complaint, we found that a limited-sequence imaging protocol can identify nearly all significant brain abnormalities with the major exceptions of small, contrast-enhancing lesions and vascular lesions that are revealed by MR angiography. The 4-sequence protocol was superior to the 3-sequence protocol due to improved detection of probable microhemorrhages, and we conclude that it is sufficiently robust for the routine evaluation of patients who have new neurological symptoms and do not have a clear clinical indication for the use of contrast (e.g. history of a primary non-CNS malignancy or suspicion of demyelinating/inflammatory disorders) or direct evaluation of the cerebral vasculature (e.g. vascular risk factors with high suspicion for acute ischemia).

The T2-weighted FLAIR sequence has been shown to be the single most sensitive sequence for the detection of brain pathology [1]–[16]. Saleh et al. demonstrated in 1026 consecutive outpatient MRI studies that this sequence alone had positive and negative predictive values of 96.1% and 98.4%, respectively, for lesions detectable by a complete MRI protocol that included contrast enhancement [17]. The data presented here are fully consistent with this work. However, the T2-weighted FLAIR sequence is not sensitive in detecting small enhancing lesions, small structural lesions, early ischemia, and microhemorrhages, and it cannot be relied upon as the only sequence. The use of diffusion MRI and susceptibility sequences are excellent supplemental sequences for the detection of the latter two types of abnormalities.

Cerebral microhemorrhages are a common finding, but have indeterminate prognostic and therapeutic significance [18]. Using the 3-sequence protocol, 78% of cases with probable cerebral microhemorrhages were undetected by readers viewing the T1, T2-weighted FLAIR and diffusion sequences. This is similar to prior reports [19], [20]. All probable microhemorrhages were correctly identified by readers who were able to view an axial susceptibility sequence as part of the 4 sequence protocol. This finding is consistent with the known superiority of susceptibility-weighted sequences in identifying small foci of susceptibility effect [18]–[23].

The use of the 3-sequence protocol resulted in lack of detection of 25% of major abnormalities, with over half of these being enhancing lesions. An improved detection rate was manifest with the 4-sequence protocol, with a lack of detection of 16% of major findings, but 90% of these were enhancing lesions. Enhancing lesions included meningiomas, active inflammatory/demyelinating lesions, metastases and probable primary brain tumors, as well as indeterminate punctate areas of parenchymal enhancement. Our findings suggest that intravenous gadolinium-based contrast should be administered in patients with a history of malignancy or suspected demyelinating/inflammatory disorders.

Several significant vascular lesions were not identified on the 3- or 4-sequence protocols, even upon revisiting the study, but were identified on MRA or MRV. Two of the missed vascular abnormalities in group 1 were occlusions in MCA branches. In cases where arterial dissection, vascular thrombosis or occlusion is clinically suspected, a vascular imaging sequence such as an MRA or MRV may add considerable value (Figures 11D-F and 12E-G).

One case of cortical dysplasia in group 1 was not initially identified, but the dysplasia was discernible in retrospect (Figure 5). The finding is subtle but visible on FLAIR images, and more obvious on T2 images. In these patients with a history of seizures, additional sequences could be added to the protocol for diagnostic imaging, including the use of high resolution, isotropic multiplanar sequences that would aid in identifying the structural abnormalities that may often be subtle.

The limited 4-sequence MRI protocol should increase efficiency and reduce scan length. The amount of time saved using the 4-sequence protocol is difficult to precisely calculate due to the variable pre-scan preparation times; however, we estimate that the time saved ranges from 5 to 25 minutes per study compared to our standard protocols. Further efficiencies in study acquisition may be possible through optimization of the limited sequence parameters. Also, substantial time savings in the interpretation of the 4-sequence MRI studies are expected since there will be fewer images per study for the neuroradiologist to review.

The 4-sequence MRI protocol will only be efficient and of value if the limited sequences allow the neuroradiologist to make a correct diagnosis with a limited rate of patient recall for additional sequences. The data in our study suggest that the patient recall rate will be very low and on the order of “misses” using the standard protocol.

A proper review of the patient's history should allow the neuroradiologist to tailor the MRI sequences to be performed for any given indication. The limited 4-sequence MRI screening protocol is only appropriate for the patient with new neurological complaints without an existing diagnosis. For example, a patient with known metastatic cancer will receive a contrast-enhanced MRI study. For patients undergoing the limited screening protocol, unexpected findings may be detected that warrant work-up with additional MR sequences. In these cases, we envision a hybrid solution. A large number of cases are monitored in ‘real-time’ by the neuroradiologist, which allows for immediate tailoring of the examination based on the findings on the limited sequences. Current technology even permits the neuroradiologist to remotely monitor cases and tailor them off-site. However, there will be some cases that cannot be monitored and such patients may require additional sequences to be performed at another time.

This study has several limitations. It has a retrospective design, although effort was made to minimize bias by analyzing consecutive cases. Despite our diligent attempt to minimize bias, results should be interpreted with caution as they have not yet been validated in a prospective fashion or using active clinical cases. A prospective study is currently being designed to confirm the findings described here. An additional limitation is that the impressions from each original MRI interpretation were used as the reference standard. There may have been findings on brain MRI that were mentioned only in the body of a report and not in the impression. This most notably occurred in patients with probable microhemorrhages. Another limitation is that the original MRI interpretations were performed in the true clinical setting where the neuroradiologist may have been interrupted or distracted by phone calls, teaching and consultations, which is different than the clinical research conditions under which the group 1 and 2 neuroradiologists reviewed the cases. However, the group 1 and 2 neuroradiologsists interpreted their cases using the same PACS workstations in the same clinical work areas as those of the original interpretations and, therefore, were subject to similar disturbances and distractions. In addition, for the original clinical interpretations, the readers also had access to the full medical record that is readily accessible at the workstation, as well as the ability to contact the referring the physician for further information. Thus the reading of the limited sequences was more restricted than the original reading, thus providing an added measure of assurance on the validity of the results. The heterogeneity of our institutions MRI scanners, which had different manufacturers and field strengths, may be considered as another limitation. Bearing that in mind, the national installed base of clinical magnets has far greater heterogeneity than our institutions and one might argue that these studies cannot reflect the diversity of clinical practice. Additionally, this paper describes an effort to limit the number of sequences required for routine performance and is not making any comment with respect to interpretation times. Finally, the readers were primarily experienced neuroradiologists, which is not necessarily true for all those interpreting brain MRIs. We have not attempted to validate error rates utilizing this protocol amongst general radiologists or other specialists.

## Conclusions

A four-sequence MRI protocol that includes sagittal T1 weighted images, axial T2-weighted FLAIR images, axial diffusion-weighted images, and T2*-weighted images reliably detects nearly all non-enhancing brain MRI findings in patients without a known history of a neurologic disease undergoing evaluation for a new neurologic complaint. If a patient has persistent unexplained symptoms, an additional MRI study including post-contrast and high-resolution imaging should reveal abnormalities characterized by blood brain barrier breakdown or small structural lesions. Clinical information suggesting a vascular lesion should be evaluated using an angiographic or venographic sequence.
